# New comparative genomics approach reveals a conserved health span signature across species

**DOI:** 10.18632/aging.100342

**Published:** 2011-06-29

**Authors:** Michael Antosh, David Fox, Stephen L. Helfand, Leon N Cooper, Nicola Neretti

**Affiliations:** ^1^ Department of Molecular Biology, Cell Biology and Biochemistry, Division of Biology and Medicine, Brown University, Providence, RI 02912, USA; ^2^ Department of Physics, Brown University, Providence, RI 02912, USA; ^3^ Institute for Brain and Neural Systems, Brown University, Providence, RI 02912, USA

**Keywords:** meta analysis, resveratrol, aging, sirtuins, dietary restriction

## Abstract

Environmental and genetic interventions extend health span in a range of organisms by triggering changes in different specific but complementary pathways. We investigated the gene expression changes that occur across species when health span is extended via different interventions. To perform this comparison using heterogeneous datasets from different measurement platforms and organisms, we developed a novel non-parametric methodology that can detect statistical significance of overlaps in ranked lists of genes, and estimate the number of genes with a common expression profile. By comparing genetic and environmental interventions that consistently lead to increased health span in invertebrates and vertebrates we built a conserved health span signature and described how such a signature depends on tissue type. Furthermore, we examined the relationship between calorie restriction and resveratrol administration and for the first time, identified common gene and pathway changes in calorie restriction and resveratrol in both invertebrates and mammals. Our approach can thus be used to explore and better define the relationships between highly complex biological phenomena, in this case those that affect the health and longevity.

## INTRODUCTION

Aging is a very complex phenotype: it can assume different forms in different species, individuals, and tissues, and its mechanisms are multiple, complex and stochastic in nature. Several interventions that extend life span in many organisms have been identified, including environmental factors, genetic manipulations and drugs. It is thought that these diverse interventions share some common pathways through which they extend life span.

Dietary restriction (DR) has been shown to consistently improve health-span in many model organisms [1, 2]. Several studies have attempted to identify genes responsible for extending lifespan through dietary restriction, but such efforts have been hindered by the overwhelming number of gene expression changes induced by the change in diet, the majority of which are unlikely to be causal factors of the longevity response. Of the genetic manipulations that are known to extend lifespan, many have been associated, at least partially, to the DR longevity pathway [1, 3-5]. In a previous study, we demonstrated that it is possible to identify common gene signatures and a novel longevity gene by comparing gene expression changes in DR and in interventions that are known to be related to DR [6]. One of the genes used in that study, Sir2 in *Drosophila melanogaster* and its mammalian homolog Sirt1, has been extensively studied in the field because of its role in reducing age-related pathologies in a wide range of organisms [7, 8]. In fruit flies Sir2 has been identified as an important mediator of DR-induced physiological and longevity responses [9, 10]. More recently, the small molecule resveratrol, a Sirt1 activator, has been shown to induce a longevity response in yeast [11], the worm and fruit fly [12], and increase the health span in mouse [13, 14]. However, the specific mechanism through which resveratrol and other small molecule activators of Sirt1 induce such beneficial effects is still controversial [11, 15-22].

In this study, we combined available gene expression profiling datasets to study the similarities in the transcriptional changes induced by DR, Sir2 overexpression and resveratrol administration in *D. melanogaster* and in the mouse. Because of the heterogeneity of the datasets used, which have been generated in different laboratories, using different protocols and analysis pipelines, we developed a novel non-parametric statistical approach to compare gene lists from different experiments. This new algorithm does not rely on the choice of a threshold for the statistical parameters used in detecting differential expression, but rather tries to gain power from the gene list in one experiment to inform gene selection in a different experiment.

## RESULTS AND DISCUSSION

### Comparing ranked lists of genes

We have developed a novel methodology to compare gene expression datasets across experiments. Differences in protocols, platforms, and number of replicate measurements can lead to significant differences in the stringency of the criteria used for detecting differential expression across experiments. The procedure most often used is to analyze the data and set an arbitrary *p* value cutoff for statistical significance and fold-change, then select sets of genes separately in the different datasets and compare the different sets to find the genes in their intersection. However, it is possible that the dynamic range in the different experiments is different, or that the experimental protocol has introduced more or less variance among replicates. We reasoned that if two gene lists are really related to each other because they share a large number of common genes, we could use the information in one list to inform the detection of significant genes in the other. This will then serve two purposes: to improve on the number of genes that are deemed significantly affected by the treatment, and at the same time to assess the degree of overlap between two experiments.

We used a non-parametric approach to compare lists of genes based on the rank statistics, where genes were ranked according to the magnitude of the change in expression between the treatment and control conditions. A similar approach has been successfully applied to the study of enrichment of functional categories of genes [23]. Starting from the genes with the highest ranks in the two conditions we want to compare, we looked at the probability that the observed overlap between such genes could be explained by chance alone. We then marched down the ranks in steps by adding genes until any of the following two criteria was not met: 1) the new overlap was not statistically significant; 2) the increase in the overlap from the previous step could be explained by chance alone. A detailed statistical derivation of the comparison method is given in the Supplemental Information.

**Figure 1:**
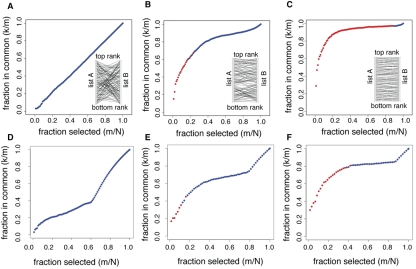
Statistical significance of the overlap between gene lists. Each panel shows the fraction of genes in common between two lists of genes (*k/m*) as a function of the fraction of genes selected in each list (*m/N*). The overlaps selected as statistically significant by the list comparison algorithm are marked in red. The top three panels correspond to simulated data, with the inlets representing how list A of genes was shuffled to generate list B. (**A**) The two lists are completely unrelated: list A was randomly shuffled to generate list B. (**B**) The two lists are partially related: list A was shuffled with a constrain on the number of ranks a gene could jump to. (**C**) The two lists are strongly related: same shuffling as in (**B**), with a stricter constrain. The bottom three panels correspond to list comparisons from datasets in Bauer *et al.* [6]. (**D**) The algorithm did not detect any significant overlap when applied to genes up-regulated when Dmp53 activity is diminished in *D. melanogaster*, and up-regulated genes in the yw, w1118 strain with respect to the Canton-S strain used. (**E**) The algorithm detected a significant overlap between up-regulated genes in the DR and Sir2 datasets. (**F**) The algorithm detected a large overlap between DR in two different fly strains (yw, w1118 and Canton-S).

First, we evaluated our method on simulated data generated as follows. We took a ranked list of genes (list A) and generated a new dataset by completely scrambling the list order (list B) (Figure 1A), such that the two lists would have no association between them. The inlet panel in Figure 1A shows the scrambling process. Then, we computed the percent overlap between the top 100 ranking genes in the two lists and repeated the process by adding 100 genes at a time from the ranked lists. Every time we added genes, we tested the statistical significance of the overlap and marked significant overlaps with red dots; we represented overlaps that could have been obtained by chance alone with blue dots. The two lists do not show any significant overlap for any of the ranks, because, as expected, the proportion of genes in the overlaps (y-axis) is roughly equal to the proportion of genes selected (x-axis).

Then, we simulated and tested the case in which two lists are related to each other. The rank of genes in list A was shuffled to generate list B, but this time we limited the number of ranks a given gene could jump to (see inlet in Figure 1B). In this case, the algorithm detected a significant overlap between the two lists (Figure 1B). When we limited the number of ranks a gene could change to between the two lists (inlet in Figure 1C) such that the two lists were very similar to each other, the algorithm selected a very large overlap (Figure 1C).

We then tested the algorithm on real data, using three different combinations of datasets from Bauer *et al.* [6]. In the first example, we compared the ranked gene list from decreased Dmp53 activity to a ranked list created by examining the differences in gene expression between two of the control strains used in the DR experiments in that study, specifically the yw, w^1118^ and the Canton-S strains on high calorie (1.5N) food. Changes in expression induced by a gene mutation that extends lifespan are unlikely to be related to differences associated with specific genetic backgrounds.

Consistently, our algorithm did not detect any significant overlap between them (Figure 1D). However, when we compared the DR and the Sir2 datasets, we detected a significant overlap between the two lists (Figure 1E), as reported before [6]. As expected, the degree of overlap was even better when we compared the effect of the same intervention, DR, in two different fly strains (Figure 1F).

### Life span extension in *Drosophila melanogaster*

Life span extension can be achieved by environmental interventions, genetic manipulations and drugs, and some longevity pathways are thought to be shared by these three modalities. One example is the small mole- cule resveratrol, which is believed to mimic the beneficial effects of dietary restriction by enhancing Sirt1 activity, especially in *D. melanogaster* and *C. elegans* [12]. We combined several available expression array datasets and used the list comparison algorithm to compare gene lists corresponding to DR, Sir2 overexpression and resveratrol treatment. Hence, the collection of experimental data we worked with contained data from different interventions, genetic backgrounds and tissues. Table I shows a breakdown of the datasets we used in *D. melanogaster*. Each dataset corresponds to a treatment/control pair of expression microarrays sets, with each set containing three biological replicates for both treatment and control. In what follows, overlaps between lists are reported if and only if such overlaps reached statistical significance. The complete list of genes identified using the list comparison algorithm can be found in the Supplemental Data.

**Table 1:** Drosophila datasets used in comparison analysis

Intervention	Genetic background	Tissue	Source
Dietary restriction	Canton-S	head/thorax	Antosh *et al.* [30]
Dietary restriction	Canton-S	whole body	Bauer *et al.* [6]
Dietary restriction	yw, w^1118^	whole body	Bauer *et al.* [6]
Sir2 overexpression	yw, w^1118^	whole body	Bauer *et al.* [6]
resveratrol	Canton-S	head/thorax	Antosh *et al.* [30]

**Figure 2:**
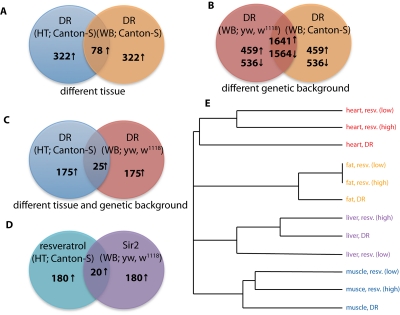
Comparison of different longevity interventions in *D. melanogaster*. (**A**) The Venn diagram generated by the list comparison algorithm when comparing DR in the same fly strain (Canton-S) in two different tissues (WB = whole body versus HT = head-thorax) shows a relatively small intersection (78 up-regulated genes). (**B**) A much larger intersection was detected when comparing DR in two different fly strains (yw, w1118 and Canton-S) but in the same tissue (1641 up-regulated and 1564 down-regulated genes). (**C**) Consistently, a very small overlap was detected when the comparison was done across tissues and fly strains (25 up-regulated genes). (**D**) A significant overlap of 20 up-regulated genes was found between the resveratrol and Sir2 datasets, despite the two datasets used different genetic backgrounds and tissues. (**E**) Hierarchical clustering of the different mouse gene expression datasets from Pearson *et al.* [17], which include two levels of resveratrol treatment (low and high), DR and four tissues: heart, white adipose tissue, liver and skeletal muscle. The clustering reveals a strong tissue effect: different interventions in the same tissue are clustered together.

We first assessed the effect of different genetic backgrounds and tissues on the same treatment. While only 78 genes (Figure 2A) were selected in the intersection between DR datasets from different tissues - whole body versus head/thorax - a much larger number, 3,205 (Figure 2B) were shared by DR fruit flies from different genetic backgrounds (Canton-S and yw, w^1118^). Hence, the tissue effect appears to have a predominant role in determining the set of genes responding to a longevity intervention. As expected, direct comparison of DR between datasets where both genetic background and tissue were different, revealed an even smaller overlap of only 25 genes (Figure 2C).

When we compared the Sir2 dataset with the resveratrol dataset, there were a significant number of genes (20) shared by the two longevity interventions (Figure 2D). Although this number was relatively small, the two datasets differed in both genetic background and tissue. If we take that into account and compare this intersection with the number of genes in common between the two DR datasets in Figure 2C, the small number likely reflects an underestimate of the true overlap between the significant genes in the two interventions.

Of the 20 genes in the intersection, 9 were annotated as belonging to the proteolysis gene ontology (GO:0006508). One of the genes, ninaD is involved in phototransduction and recently its mammalian homolog, the scavenger receptor SR-BI [24], has been shown to have a role in Alzheimer's disease pathogenesis [25].

### Dietary restriction and resveratrol in mice

We next looked at a similar dataset from mice that reproduced, at least partially, our Drosophila dataset. Pearson et al. [17] have studied the expression changes induced by DR and two levels of resveratrol (high and low) in four different mouse tissues: heart, white adipose tissue, liver and skeletal muscle. The dataset however did not contain Sirt1 overpression and, to our knowledge, such a dataset has not been published yet. We applied our novel list comparison algorithm to all pair-wise comparisons between the treatment/control pairs in this dataset and clustered interventions using a hierarchical clustering algorithm with a metric based on the degree of overlap between lists (see Material and methods). The dendrogram resulting from this clustering procedure is shown in Figure 2E. The degree of similarity between interventions within the same tissue was higher than the degree of similarity between the same interventions across tissues. In fact, datasets were grouped by tissue first (same color in figure), and by treatment second, with the exception of liver, in which DR and high resveratrol were more similar to each other than to the low resveratrol. Although the mouse data did not contain expression profiles for different genetic backgrounds, the strong tissue effect displayed by the clustering algorithm confirms our previous findings in *D. melanogaster*.

Our findings are consistent with a good deal of experimental data indicating resveratrol is a DR mimetic in mammals and flies [12, 13, 17, 26, 27]. For example, Berger et al. [27] reported that in three different tissues - heart, skeletal muscle and brain - the expression profile induced by a low dose of dietary resveratrol partially mimics the DR expression profile. Consistently, when we applied the list comparison algorithm to this dataset we detected in all three tissues a large and significant overlap between genes responding to both DR and resveratrol with changes in the same direction (heart: 977 genes; skeletal muscle: 1,733 genes; brain: 912 genes). These sets of genes overlapped, at least in part, with the sets we identified in Pearson et al. [17], although the overlaps did not reach statistical significance.

### Conserved health span signature between invertebrates and mammals

**Figure 3:**
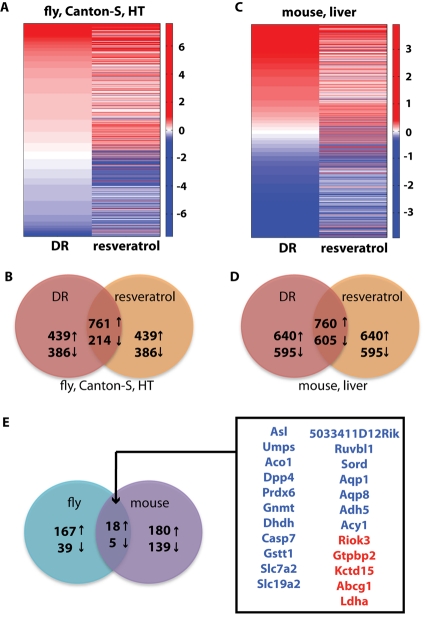
Comparison of dietary restriction and resveratrol treatment in fruit fly and mouse. (**A**) Heat map of the fold-changes of the genes selected by the list comparison algorithm in either the DR or resveratrol dataset in the head-thorax (HT) of Canton-S female fruit flies. (**B**) The Venn diagram of the genes selected in the comparison between DR and resveratrol in the fly dataset shows a significant intersection of 975 genes. (**C)** Heat map of the fold-changes of the genes selected by the list comparison algorithm in either the DR or resveratrol dataset in the mouse liver dataset from Pearson *et al.* [17]. (**D**) The Venn diagram of the genes selected in the comparison between DR and resveratrol in the mouse liver dataset shows a significant intersection of 1,365 genes. (**E**) Venn diagram of the comparison between the DR/resveratrol signature in fly and mouse. The 23 genes in common are listed and color-coded in blue (up-regulated) and red (down-regulated). The 229 fly genes and 342 mouse genes used in this comparison were selected out of the 975 and 1,365 respectively as having a homolog gene in the other species.

We wanted to determine if our new algorithm could detect a DR/resveratrol signature shared across species. First, we compared the gene list from DR to the gene list from resveratrol in Drosophila to define a resveratrol/DR signature. This identified 975 genes changing in the same direction in both DR and resveratrol treatment in fruit flies (761 up-regulated and 214 down-regulated) (Figures 3A and 3B). In mice, a resveratrol/DR signature identified 1,365 genes changing in the same direction, with 760 up-regulated and 605 down-regulated (Figures 3C and 3D). Then, we matched genes across species using the HomoloGene database (see Materials and methods). Of the 975 genes in the Drosophila signature, 229 genes had a mouse homolog according to the HomoloGene database, while of the 1,365 genes in the mouse signature, 342 had a fruit fly homolog. We then compared the direction of change of these genes in the two species and identified 18 genes that were up-regulated in both and 5 genes that were down-regulated in both (Figure 3E).

Of the 23 genes in common between fly and mouse, 9 (39%) are involved in stress response functions such as response to starvation (Asl and Sord), oxidative stress (Aco1, Prdx6, Dhdh, Gstt1, Ldha and Acy1) and cellular protection (Ruvbl1), while 8 (35%) are involved in metabolism and growth (Umps, Dpp4, Gnmt, Slc7a2, Slc19a2, Riok3, Gtpbp2 and Acy1). Response to stress, oxidative stress in particular, has been identified as one of the possible mediators to the longevity response [28]. The intersection also contained two aquaporins, both up-regulated in all health span-extending interventions we studied. Interestingly, Lee *et al.* have recently shown that in *C. elegans* glucose shortens lifespan by down-regulating the expression of an aquaporin gene [29].

### Conclusions

Our findings demonstrate it is possible to identify preserved gene signatures by pair-wise comparison of gene lists from different health span extending interventions. The list comparison method we have developed can identify shared genes as well as the degree of similarity between two interventions. We have shown that both genetic background and predominantly tissue play an important role in defining a signature. However, our novel algorithm was sufficiently sensitive to detect common genes across different tissues and genetic backgrounds.

By comparing gene expression changes induced by Sir2 overexpression and resveratrol in *D. melanogaster* we have shown for the first time a significant overlap between the two datasets. This is consistent with a model in which Sir2 and resveratrol share a common longevity pathway. The overlap, while significant, was relatively small; however, this could be explained by the fact that the two datasets, Sir2 and resveratrol, were collected using different tissues and genetic backgrounds. Because our results show that these differences can drastically reduce the size of the overlap, it is likely that the intersection we detected underestimates the true size of the overlap. It would be very interesting to know if and how many of these genes are shared by Sirt1 overexpression and resveratrol treatment in mice. However, no Sirt1 overexpression dataset in mouse is currently available.

Our novel gene expression dataset of DR and resveratrol treatment in *D. melanogaster* has revealed a large degree of similarity in the gene expression response to the two longevity interventions. In addition, by applying the list comparison algorithm to a similar dataset from mouse, we confirmed and extended the findings already reported in [17] about the close association of the two interventions in mouse. Finally, by comparing homologous genes across species we identified a preserved signature comprised of 23 genes that change in the same direction in response to DR and resveratrol treatment in both *D. melanogaster* and mouse, the majority of which are involved in stress response, metabolism and growth.

## MATERIAL AND METHODS

### Gene Expression datasets

The *D. melanogaster* dataset was obtained by combining gene expression datasets from [17] and [30]; mouse gene expression data were obtained from [6]. GCRMA [31] was used for quantile normalization and summarization of the data to generate expression scores in the log_2_ scale. Probesets with an expression score below the 25^th^ percentile (compared to the rest of the mean expressions for that condition) in both treatment and control were removed from further analysis. Fold-changes used in the list comparison algorithm were computed as the ratio between the average expression scores of the three biological replicates in the treatment and control cohorts. The fold-change of genes with more than one probeset on the array were computed as the average fold-change between all corresponding pobesets.

### Comparison of gene lists

The probability of observing at least k* genes in the intersection of two lists generated by randomly choosing two sets of *m* genes out of a total of *N* was computed as:

$$P(X\geq k^{*})=\sum_{k=k^{*}}^{m}P(X=k)$$

where

$$P(X=k)=\frac{\left(\begin{array}{c} m\\ k \end{array}\right)\left(\begin{array}{c} N-m\\ m-k \end{array}\right)}{\left(\begin{array}{c} N\\ m \end{array}\right)}$$

is the hypergeometric distribution. A mathematical derivation of the list comparison algorithm can be found in the Supplementary Information. R code to run the list comparison algorithm and its documentation is available at http://www.physics.brown.edu/physics/researchpages/ibns/listcomp.htm.

### Clustering

Hierarchical clustering was performed using a complete linkage algorithm and using *m_ij_* = max_(*l,m*)=1…*N*_ (*k_lm_*) − *k_ij_* as metrics, where *N* is the total number of experimental conditions to be compared, and *k_i_*_j_ is the number of genes in the intersection when comparing the *i*-th experimental condition to the *j*-th experimental condition.

Probesets identification numbers in the fruit fly and mouse microarrays were converted to the corresponding gene symbol and matched across species using the HomoloGene database from NCBI.

## SUPPLEMENTAL MATERIAL



## References

[R1] Guarente L, Picard F (2005). Calorie restriction–the SIR2 connection. Cell.

[R2] Kenyon C (2005). The plasticity of aging: insights from long-lived mutants. Cell.

[R3] Kapahi P (2004). Regulation of lifespan in Drosophila by modulation of genes in the TOR signaling pathway. Curr Biol.

[R4] Panowski SH (2007). PHA-4/Foxa mediates diet-restriction-induced longevity of C. elegans. Nature.

[R5] Wang PY (2009). Long-lived Indy and calorie restriction interact to extend life span. Proc Natl Acad Sci U S A.

[R6] Bauer J (2010). Comparative transcriptional profiling identifies takeout as a gene that regulates life span. Aging.

[R7] Herranz D, Serrano M (2010). SIRT1: recent lessons from mouse models. Nat Rev Cancer.

[R8] Donmez G, Guarente L (2010). Aging and disease: connections to sirtuins. Aging Cell.

[R9] Bauer JH (2009). dSir2 and Dmp53 interact to mediate aspects of CR-dependent lifespan extension in D. melanogaster. Aging.

[R10] Parashar V, Rogina B (2009). dSir2 mediates the increased spontaneous physical activity in flies on calorie restriction. Aging.

[R11] Howitz KT (2003). Small molecule activators of sirtuins extend Saccharomyces cerevisiae lifespan. Nature.

[R12] Wood JG (2004). Sirtuin activators mimic caloric restriction and delay ageing in metazoans. Nature.

[R13] Baur JA (2006). Resveratrol improves health and survival of mice on a high-calorie diet. Nature.

[R14] Hofseth LJ (2010). Taming the beast within: resveratrol suppresses colitis and prevents colon cancer. Aging.

[R15] Kaeberlein M (2005). Substrate-specific activation of sirtuins by resveratrol. J Biol Chem.

[R16] Milne JC (2007). Small molecule activators of SIRT1 as therapeutics for the treatment of type 2 diabetes. Nature.

[R17] Pearson KJ (2008). Resveratrol delays age-related deterioration and mimics transcriptional aspects of dietary restriction without extending life span. Cell Metab.

[R18] Beher D (2009). Resveratrol is not a direct activator of SIRT1 enzyme activity. Chem Biol Drug Des.

[R19] Pacholec M (2010). SRT1720, SRT2183, SRT1460, and resveratrol are not direct activators of SIRT1. J Biol Chem.

[R20] Dai H (2010). SIRT1 activation by small molecules: kinetic and biophysical evidence for direct interaction of enzyme and activator. J Biol Chem.

[R21] Morselli E (2009). Autophagy mediates pharmacological lifespan extension by spermidine and resveratrol. Aging.

[R22] Armour SM (2009). Inhibition of mammalian S6 kinase by resveratrol suppresses autophagy. Aging.

[R23] Mootha VK (2003). PGC-1alpha-responsive genes involved in oxidative phosphorylation are coordinately downregulated in human diabetes. Nat Genet.

[R24] Voolstra O (2006). The Drosophila class B scavenger receptor NinaD-I is a cell surface receptor mediating carotenoid transport for visual chromophore synthesis. Biochemistry.

[R25] Thanopoulou K (2010). Scavenger receptor class B type I (SR-BI) regulates perivascular macrophages and modifies amyloid pathology in an Alzheimer mouse model. Proc Natl Acad Sci U S A.

[R26] Barger JL (2008). Short-term consumption of a resveratrol-containing nutraceutical mixture mimics gene expression of long-term caloric restriction in mouse heart. Exp Gerontol.

[R27] Barger JL (2008). A low dose of dietary resveratrol partially mimics caloric restriction and retards aging parameters in mice. PLoS One.

[R28] Vermeulen CJ, Loeschcke V (2007). Longevity and the stress response in Drosophila. Exp Gerontol.

[R29] Lee SJ, Murphy CT, Kenyon C (2009). Glucose shortens the life span of C. elegans by downregulating DAF-16/FOXO activity and aquaporin gene expression. Cell Metab.

[R30] Antosh M (2011). Comparative transcriptional pathway bioinformatic analysis of dietary restriction, Sir2, p53 and resveratrol life span extension in Drosophila. Cell Cycle.

[R31] Wu Z (2004). A Model-Based Background Adjustment for Oligonucleotide Expression Arrays. Journal of the American Statistical Association.

