# Supplementary material for: New comparative genomics approach reveals a conserved health span signature across species
Source: Aging (Albany NY). 2011 Jun 29;3(6):576–83. doi: 10.18632/aging.100342 (PMC3164366; doi:10.18632/aging.100342)
Supplement: Supplementary file 1 [file aging-03-576-s001.pdf]

Supplemental Information

1 List comparison algorithm: problem formulation

The goal of the gene list comparison algorithm is to assess how similar two set of significant genes obtained from two experiments are. Genes are ranked according to a chosen metric, for example the fold-change between treatment and control in each experiment. We will refer to the two lists of genes generated in this manner as list A and list B. The first step is to compute the statistical significance of the intersection between two sets of genes from the two lists.

2 Statistical significance of ranked lists intersection

Table 1 shows the contingency table describing the comparison of the top m genes across two experimental conditions. A and B are the two experimental conditions, N is the total number of genes, m is the number of genes selected from each experiment (typically the top m genes from the ranked list are selected), and k is the number of genes in the intersection.

Since the margins of Table 1 are fixed, the probability of observing k genes in the intersection of two lists generated by randomly choosing two sets of m genes out of a total of N is given by the hypergeometric distribution:

$$P(X = k) = \frac{\binom{m}{k}\binom{N-m}{m-k}}{\binom{N}{m}} \tag{1}$$

Table 1: Contingency table describing the selection of the top m genes.

	A	$\bar{A}$	Total
B	k	$m-k$	m
$\bar{B}$	$m-k$	$N+k-2m$	$N-m$
Total	m	$N-m$	N

Table 2: Contingency table for the selection of additional $m_2 - m_1$ genes.

	A	$\bar{A}$	Total
B	$k_2 - k_1$	$m_2 - k_2$	$m_2 - k_1$
$\bar{B}$	$m_2 - k_2$	$N + k_2 - 2m_2$	$N - m_2$
Total	$m_2 - k_1$	$N - m_2$	$N - k_1$

If k^* is the observed number of genes in the intersection, we want to compute the probability of observing at least k^* genes in the intersection when the two lists are randomly generated. This probability is given by:

$$P(X \geq k^*) = \sum_{k=k^*}^{m} P(X = k) \tag{2}$$

3 Statistical significance of adding genes

When we increase the number of genes we compare in the two lists, i.e. the value of m, the number of genes in the intersection is bound to increase as well. It is possible that although the intersection is still significant, the number of genes we added to the intersection is close to what we would expect from random chance. In order to test for this we need to compute such probability. Let k_1 be the number of genes in the intersection when we select m_1 genes from the two lists, and k_2 the number of genes in the intersection when we select m_2 genes.

The probability of observing the values in Table 2:

$$P(X = k) = \frac{\binom{m_2-k_1}{k_2-k_1}\binom{N-m_2}{m_2-k_2}}{\binom{N-k_1}{m_2-k_1}} \tag{3}$$

The probability of observing an intersection larger or equal to k^* is then:

$$P(X \geq k^*) = \sum_{k=k^*}^{m_2} \frac{\binom{m_2-k_1}{k-k_1}\binom{N-m_2}{m_2-k}}{\binom{N-k_1}{m_2-k_1}} \tag{4}$$

So for a given $\Delta_k = k_2 - k_1$ it is possible to use Equation (4) to test for significance of the intersection between the two lists for the added $\Delta_m = m_2 - m_1$ genes in the ranked list. Specifically, adding Δ_m genes leads to a significant increase to the intersection between the two lists if $P(X \geq \Delta_k) \leq \alpha$ where α is the significance level.

4 List comparison algorithm

The top ranking m genes in the two experimental conditions are compared by computing the two probabilities in Equations (2) and (4). The value of m is

increase with a step Δ_m until either of the two probabilities is larger than a set significance value α. In the analysis of both simulated and experimental data in this paper we used $\Delta_m = 100$ and $\alpha = 0.05$. In the experimental datasets, the algorithm was applied separately to both up-regulated and down-regulated genes. For the down-redulated gene, genes were ranked in increasing order of fold-change, i.e. from the largest negative fold-change to the smallest negative fold-change.

5 Algorithm performance

We evaluated the performance of the gene list comparison method on simulated data generated as follows. For any given value of m and k, we took a ranked list of $N = 10,000$ genes (list A) and randomly selected a set of k genes among the top m genes. These k genes were distributed randomly across the top m ranks of list B. All of the remaining genes in list A were randomly distributed among the remaining ranks of list B, from 1 to N, that had not already been occupied by the initial k genes. This procedure guaranties that the two lists will have at least k genes in common among the top m genes. We then applied the list comparison algorithm as described in Section 4 for different values of m and k to empirically estimate the value of m. For each value of m and k we computed the percent of times out of 100 simulations the estimated of m was equal to the original value used to generate the data. Figure 1 shows this percentage as a function of k/m for different values of m/N, from 4% to 15%. When just 20% of the genes are in common among the top 4% of the total number of genes in the lists, the algorithm estimated the correct value of m over 80% of the times, and reached a 100% when 40% or more genes were in common. As we increased the value of m in the simulations, the value of k/m needed to achieve a percentage of correct estimation of 80% or more increased. This is expected because the statistical significance of the overlap decreases for increasing values of m.

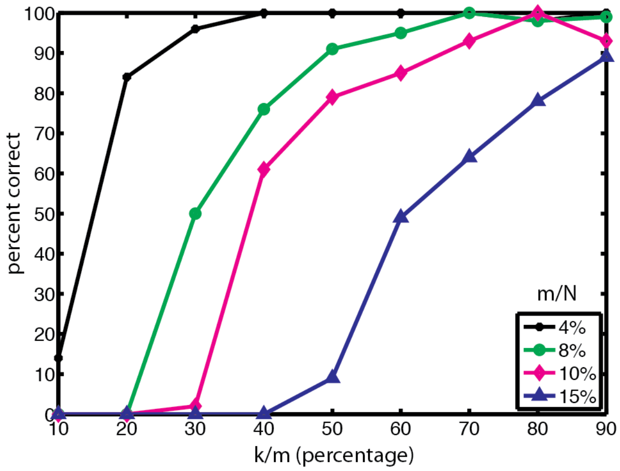

Figure 1: Performance of the list comparison algorithm on simulated data. Two lists with a known number k of common genes among the top m genes in the rank ordered list were analyzed using the list comparison algorithm. The y-axis corresponds to the percentage of times the correct value of m was estimated out of 100 simulations.
